# New Medical Journals

**Published:** 1866-06

**Authors:** 


*New Medical Journals.*—The first number of the "Southern Journal of Medical Sciences," of New Orleans, is on our table, and is entered with great pleasure on our list of exchanges. We had the pleasure of its perusal, however, saddened by the astounding news of the death of its leading editor, Dr. E. D. FENNER. We wish the Journal all the success marked out for it by its lamented senior editor.

The third series of the "Southern Medical and Surgical Journal," at Augusta, Ga., will be commenced the first of July next, edited by JOSEPH JONES, M. D., Professor of Chemistry in the Medical College of Georgia. It will be issued bi-monthly, and contain one hundred and seventy-six pages. Terms $5.00 per annum.

New series of the "Nashville Journal of Medicine and Surgery," a monthly of eighty pages, will be issued the first of July. Edited by W. K. BOWLING, M. D., Professor of Practice, assisted by P. F. EVE, M. D., Professor of Surgery, University of Nashville. Subscription price $5.00.

The announcement of the fifty-ninth session of the School of Medicine, in the University of Maryland, commencing 15th October, 1866, is received. In addition to the full corps of eight regular Professors, five adjunct Professors to the chairs of Surgery, Obstetrics, Practice, Materia Medica and Chemistry are announced. The course will end on the first of March, 1867, four months and a half being occupied, instead of four, as heretofore.

Address GEO. W. MILTENBERGER, M. D.,
Dean of Faculty.

